# The association between gene variants and longitudinal structural brain changes in psychosis: a systematic review of longitudinal neuroimaging genetics studies

**DOI:** 10.1038/s41537-017-0036-2

**Published:** 2017-11-01

**Authors:** Julia H. Harari, Covadonga M. Díaz-Caneja, Joost Janssen, Kenia Martínez, Bárbara Arias, Celso Arango

**Affiliations:** 1 Department of Child and Adolescent Psychiatry, Hospital General Universitario Gregorio Marañón, CIBERSAM, Instituto de Investigación Sanitaria Gregorio Marañón (IiSGM), School of Medicine, Universidad Complutense, Madrid, Spain; 2 University of Wisconsin School of Medicine and Public Health, Madison, WI USA; 3 Department of Psychiatry, Brain Center Rudolf Magnus, University Medical Center Utrecht, Utrecht, The Netherlands; 4 Zoology and Biological Anthropology Unit. Departament de Biologia Evolutiva, Ecologia i Ciències Ambientals. IBUB., Faculty of Biology, Universitat de Barcelona, Barcelona, Spain; 5 CIBERSAM (Centro de Investigación Biomédica en Red de Salud Mental), Instituto de Salud Carlos III, Madrid, Spain

## Abstract

Evidence suggests that genetic variation might influence structural brain alterations in psychotic disorders. Longitudinal genetic neuroimaging (G-NI) studies are designed to assess the association between genetic variants, disease progression and brain changes. There is a paucity of reviews of longitudinal G-NI studies in psychotic disorders. A systematic search of PubMed from inception until November 2016 was conducted to identify longitudinal G-NI studies examining the link between Magnetic Resonance Imaging (MRI) and Diffusion Tensor Imaging (DTI)-based brain measurements and specific gene variants (SNPs, microsatellites, haplotypes) in patients with psychosis. Eleven studies examined seven genes: BDNF, COMT, NRG1, DISC1, CNR1, GAD1, and G72. Eight of these studies reported at least one association between a specific gene variant and longitudinal structural brain changes. Genetic variants associated with longitudinal brain volume or cortical thickness loss included a 4-marker haplotype in G72, a microsatellite and a SNP in NRG1, and individual SNPs in DISC1, CNR1, BDNF, COMT and GAD1. Associations between genotype and progressive brain changes were most frequently observed in frontal regions, with five studies reporting significant interactions. Effect sizes for significant associations were generally of small or intermediate magnitude (Cohen’s *d* < 0.8). Only two genes (BDNF and NRG1) were assessed in more than one study, with great heterogeneity of the results. Replication studies and studies exploring additional genetic variants identified by large-scale genetic analysis are warranted to further ascertain the role of genetic variants in longitudinal brain changes in psychosis.

## Introduction

Psychotic disorders comprise a subset of psychiatric illnesses associated with severe consequences for affected individuals and a high global burden of disease.^1^ In these illnesses, genetic and environmental risk factors may interact with each other and with age-dependent neurodevelopmental processes to affect illness pathology and prognosis.^2–4^ Longitudinal studies are particularly important as they allow for probing the interaction between risk factors and brain changes. Meta-analyses and reviews of longitudinal studies in adult-onset schizophrenia samples have demonstrated a great variability in results, even between studies using cohorts with similar characteristics.^5,6^ The most consistent results across studies are a progressive decline in cortical gray matter (GM) volume and progressive increases in lateral ventricle (LV) volume in patients when compared to healthy controls.^5–8^ Similarly, a recent meta-analysis of early-onset psychosis (EOP, first appearance of psychotic symptoms before the age of 18 years) reported a significant progressive decrease in frontal GM volume in patients compared to controls during the first years following onset of psychotic symptoms.^9^ However, results between individual EOP studies are heterogeneous,^10,11^ and some studies of psychotic cohorts report no progressive structural changes compared to healthy controls.^12,13^

The striking disparities between published results of longitudinal neuroimaging (NI) studies may be partially explained by the effect of genetic variation on brain changes. Schizophrenia (SCZ) demonstrates high heritability,^14,15^ with a significant portion of heritability attributable to the cumulative effect of many single nucleotide polymorphisms (SNPs).^16^ Indeed, genetic association studies (including studies of previously-specified candidate SNPs, genome-wide association studies [GWAS], and studies using polygenic scores) have begun to identify SNPs in candidate genes that may contribute to the risk of developing a psychotic disorder.^16,17^

Genetic Neuroimaging (G-NI) studies analyze genetic variants and brain imaging measures in the same cohort, allowing for the assessment of associations between geno- and phenotype.^18–20^ Large-scale analyses by collaborative consortiums such as Enhancing Neuro Imaging Genetics through Meta-Analysis (ENIGMA) have suggested that genetic variation may impact structural measures such as intracranial volume (ICV) and various subcortical volumes across healthy and patient cohorts.^20,21^ In patients with psychotic disorders, genetic variants in certain genes (BDNF, COMT, RSG4, and ZNF804A, among several others) have been associated with average differences in GM and white matter (WM) brain volume, ventricular volume, and cortical thickness and folding patterns between patient genotype groups or patient vs. control genotype groups.^22,23^ However, the majority of published G-NI studies employed a cross-sectional design, which excludes the possibility of evaluating potential interactions between genetic measures and structural brain changes over time. This limitation precludes any conclusion about whether and which gene variants are associated with atypical brain changes in individuals with psychotic disorders.

Previous reviews of G-NI studies in psychosis have either covered SNPs and neuroimaging in cross-sectional studies with no or only a few longitudinal studies mentioned;^22,24–29^ have limited the scope to one or two candidate genes;^23,30,31^ or have geared the focus toward the description and use of specific neuroimaging methods rather than the integration and analysis of study results.^32,33^ Since characterizing whether and which genetic variants contribute to structural brain changes in psychosis is deemed highly relevant to the overall understanding of the disease, and many future studies will likely employ a longitudinal design, a review of longitudinal G-NI studies to date is warranted. Therefore, a systematic review was performed with the goal of synthesizing current knowledge of the association between genetic variants and progressive brain changes in psychotic disorders.

## Results

### Subject characteristics

Of the eleven studies selected for review, five studies were conducted specifically in patients experiencing a first episode of psychosis (FEP).^34–38^ Three studies evaluated patients with childhood-onset schizophrenia (COS).^39–41^ Two studies were conducted in patients with recent-onset schizophrenia spectrum disorders (recent-onset SSD) (duration of illness < 5 years), of which the percentage of FEP patients was not specified.^42,43^

Patient cohort size at baseline ranged from 58 to 119 participants. Gender distribution ranged from 58 to 79% male, and mean AAO from 10 to 30.6 years old. Eight of the eleven studies included a healthy control (HC) group for genetic,^37,42,43^ structural MRI, or both types of analysis,^34–36,40,41,44^ and one of these studies also included a sibling comparison group.^41^

Treatment with antipsychotic (AP) medication was described in eight studies.^34–38,42–44^ Ethnic distribution of the samples was either all Caucasian,^35–38,44^ 95% Caucasian,^42,43^ or 50–72% Caucasian with the remaining portion accounted for by African American, Hispanic, and Asian participants, and participants of mixed ethnicities.^34,39–41^ Additional details regarding clinical characteristics of the study samples can be found in Supplementary Table 1.

### Genetic measures

Genes examined were BDNF,^34,35,42,44^ COMT,^41^ NRG1,^36,40^ DISC1,^38^ CNR1,^37^ GAD1,^39^ and G72.^43^ All studies genotyped individual SNPs, three studies additionally performed haplotype analysis,^39,40,43^ and one study analyzed a microsatellite.^40^ Information about genes, specific variants, and descriptions of function can be found in Table 1.

**Table 1:** Genes and specific variants assessed in the genetic neuroimaging studies included in the review

Gene	Chromosome	Protein	Function	Polymorphisms included in this review	SNP Location	Functional consequences of the SNP	References
*BDNF*	11p13	Brain derived neurotrophic factor	Neurotrophin that plays a role in neurodevelopmental processes, synaptic and cognitive plasticity, and neurotransmission	Val66Met (rs6265)	Exon 2	Met allele associated with impaired secretion and lower distribution of BDNF protein in neurons	Buckley et al.;^71^ Chen et al.;^72^ Egan et al.^73^
*NRG1*	8p12-21	Neuregulin 1	Mediates cell-cell signaling, plays a role in neural development, neurotransmission and synaptic plasticity	SNP8NRG221533 (rs35753505)	Intronic, 5’ flanking region	Unknown	Harrison and Weinberger;^74^ Stefansson et al.^75^
				Microsatellite: 420M9-1395	5’ region	Unknown	
*DISC1*	1q42.1	Disrupted in schizophrenia 1	Neurodevelopment and synaptic regulation	Leu607Phe (rs6675281)	Exon 9	Allele Phe607 and Cys704 affect expression of alternative transcripts of DISC1 that are expressed in early human brain development	Hennah and Porteus;^76^ Nakata et al.^77^
				Cys704Ser (rs821616)	Exon 11		
*CNR1*	6q14-15	Cannabinoid receptor 1	GPCR for cannabinoids, mediates cannabinoid-induced CNS effects by modulating synaptic function and neurotransmission	rs1049353	Exon 4	Unknown	Castillo;^82^ Parsons and Hurd^83^
				rs2023239	Intron 2	Unknown	
*GAD1*	2q31.1	GAD67 (glutamic acid decarboxylase)	Enzyme that catalyzes GABA synthesis in inhibitory neurons	rs2270335	Intron 1	Unknown	Blum and Mann;^78^ Straub et al.^79^
				rs2241165	Intron 2	Unknown	
*G72*	13q32-34	D-amino acid oxidase activator	May regulate glutamate neurotransmission through interaction with DAAO. One isoform may play a role in mitochondrial function	rs3916965	5’ region	NA (sample divided and analyzed by haplotype, not individual SNPs)	Chumakov et al.;^80^ Kvajo et al.^81^
				rs3916967	5’ region		
				rs2391191 (Arg30Lys)	Exon 2		
				rs778294	Intron 3		
				Hap 1: AGAG			
				Hap 2: GAGG			
				Hap 3: GAGA			
*COMT*	22q11	Catechol-O-methyl transferase	Enzyme that plays a role in the breakdown of catecholamines, including dopamine	Val158Met (rs4680)	Exon 4	Met allele encodes a lower-activity COMT variant, leading to impaired dopamine catabolism	Chen et al.;^72^ Harrison and Weinberger^74^

Only SNPs and haplotypes that demonstrated an association with structural brain changes in the studies included in the review are shown

*Arg* arginine, *CNS* central nervous system, *Cys*cysteine, *DAAO* D-amino acid oxidase, *Hap* haplotype, *Leu* leucine, *Lys* lysine, *Met* methionine, *Phe* phenylalanine, *Ser* serine, *NA* not applicable, *SNP* single nucleotide polymorphism, *Val* valine

### Neuroimaging measures

No DTI studies fulfilled the complete criteria for inclusion in the review. The eleven studies identified by the search criteria used structural MRI to measure brain changes, and MRI assessments were performed on a 1.5T scanner in all studies. Further details regarding image processing software, types of sequence, slice thickness and voxel size in each study can be found in Supplementary Table 1. Eight of the studies exclusively measured volume changes, with outcomes across the studies including changes in total or regional GM or WM volume, total or regional cortical or subcortical volume (structures included the hippocampus, thalamus, caudate nucleus, and cerebellum), LV volume, and cerebrospinal fluid (CSF) volume.^34–37,39,40,42–44^ Two studies measured cortical thickness (CT) changes,^38,41^ and one study measured volume, CT, and cortical surface area (CSA) changes.^37^

### Quality assessment

Quality analysis showed that the studies included in the review were of high (64%) or moderate-high (36%) quality. Ratings for individual checklist items according to each study can be found in Supplementary Table 2.

### Genetic x structural MRI findings

Table 2 shows the main genetic x structural MRI findings from the eleven longitudinal G-NI studies retrieved. Five studies compared longitudinal brain changes between genotype or haplotype subgroups of patients with psychosis,^37–40,42,43^ while six studies compared longitudinal brain changes between genotype or haplotype groups in both patient and HC groups.^34–36,40,41,44^

**Table 2:** Main genetic neuroimaging findings

Author, Year cohort	Gene and SNP examined	Diagnosis N	Number of scans Follow-up	Morphological longitudinal measurements	Combined genetic and longitudinal NI results	EES, [95% CI]
Ho 2007^42^ Iowa Longitudinal study of recent onset schizophrenia	BDNF rs6265 (Val66Met)	Recent-onset SSD *N* = 119 (74 Val/Val; 45 Met-carriers)	2 scans 3 (1.6) years)	Frontal GM volume	Met-carriers ↓ Val/Val n.s.	0.38, [0.01, 0.75])
				Temporal GM volume	n.s.	
				Parietal GM volume	n.s.	
				Occipital GM volume	n.s.	
				LV volume	Met-carriers ↑	0.51, [0.14, 0.89]
					Val/Val n.s.	
				Sulcal CSF volume	Met-carriers ↑	0.48, [0.11, 0.86]
					Val/Val n.s.	
				Sulcal frontal CSF volume	Met-carriers ↑	≥ 0.49
					Val/Val n.s.	
				Sulcal temporal CSF volume	Met-carriers ↑	≥ 0.49
					Val/Val n.s.	
				Sulcal parietal and occipital CSF volume	n.s.	
Koolschijn 2010^44^	BDNF rs6265 (Val66Met)	SSD *N* = 87 (*N* = 68 with two scans) (44 Val/Val; 24 Met-carriers) HC *N* = 90 (*N* = 84 with two scans) (55 Val/Val; 29 Met-carriers)	2 scans (mean: SSD = 4.9 (0.5) years; HC = 4.9 (0.3) years)	Hippocampal volume	n.s.	
Smith 2012^34^	BDNF rs6265 (Val66Met)	FEP *N* = 58 (*N* = 41 with two scans) (25 Val/Val, 16 Met-carriers) HC *N* = 39 (*N* = 23 with two scans) (11 Val/Val; 12 Met-carriers)	2 scans (mean: 46 weeks)	Total brain volume Hippocampal volume	n.s.n.s.	
Suárez-Pinilla 2013^35^ PAFIP	BDNF rs6265 (Val66Met)	Non-affective FEP *N* = 80 (60 Val/Val; 20 Met-carriers) HC *N* = 54 (45 Val/Val; 9 Met-carriers)	2 scans (3 years)	Total brain volume Total GM volume Total WM volume	n.s. n.s. n.s.	
				LV volume	n.s.	
				Total CSF volume	n.s.	
				Caudate nucleus volume	n.s.	
				Thalamus volume	n.s.	
Addington 2007^40^ NIMH	NRG1 56 NRG1 markers: - 54 SNPs, 2 microsatellites - 2 erbB4 SNPs (genetic analysis only) Risk allele carriers: possession of microsatellite 420M9-1395	COS (onset before age 12) *N* = 59^a^ (75% with ≥ 2 scans) (35 risk allele carriers; 24 no risk) HC *N* = 165^a^ (65% with ≥ 2 scans) (131 risk allele carriers; 34 no risk allele)	Variable (approximately 2 years)	Total GM volumeFrontal GM volume	Greater ↓ in COS risk allele carriers than COS non-carriersn.s.	0.58 [0.05, 1.11]
				Temporal GM volume	Greater ↓ in COS risk allele carriers than COS non-carriers	0.72 [0.18, 1.25]
				Parietal GM volume	Greater ↓ in COS risk allele carriers than COS non-carriers	0.66 [0.13, 1.20]
				Occipital GM volume	Greater ↓ in COS risk allele carriers than COS non-carriers	0.59 [0.06, 1.12]
					Greater ↓ in HC risk allele carriers than HC non-carriers	0.38 [0.001, 0.76]
				Occipital WM volume	COS N.S. Greater ↓ in HC risk allele carriers than HC non-carriers	0.48 [0.10, 0.46]
				Total WM volume, WM volumes of other lobes	n.s.	
				Caudate nucleus volume	n.s.	
				Cerebellum volume	n.s.	
Suárez-Pinilla 2015^36^ PAFIP	NRG1 a) SNP8NRG221132 (rs73235619) b) SNP8NRG221533 (rs35753505) c) SNP8NRG243177 (rs6994992)	Non-affective FEP* N* = 59 (31 SNP8NRG221533 C allele-carriers; 28 T/T) HC *N* = 14 (9 SNP8NRG221533 C allele-carriers; 5 T/T)	3 scans (1 year; 2 years)	Total brain volume	n.s.	
				Total GM volume	n.s.	
				Total WM volume	SNP8NRG6221533 C allele carriers: ↓ WM volume compared to T homozygotes at 3-year scan, but no significant genotype by time interaction.	0.55 [0.03, 1.07]
				LV volume	SNP8NRG6221533 C allele carriers: 3-year ↑ compared to T homozygotes.	0.59 [0.07, 1.11]
				Total CSF volume	n.s.	
				Caudate nucleus volume	n.s.	
				Thalamus volume	n.s.	
Vázquez-Bourgon 2015^38^ PAFIP	DISC1 (a) rs6675281 (Leu607Phe) (b) rs821616 (Ser704Cys)	Non-affective FEP *N* = 79 [*N* = 63 genotyped for rs6675281 (46 Leu/Leu; 17 Phe-carriers) and *N* = 60 genotyped for rs821616 (4 Ser/Ser; 56 Phe-carriers)]	2 scans (3 years)	Total CT Frontal CT	rs6675281: Phe-carriers ↑ Leu/Leu N.S.	0.84 [0.27, 1.42]
					rs6675281 Phe + rs821616 Cys-carriers ↑	
					rs6675281 Phe-carriers ↑ Leu/Leu N.S.	0.79 [0.21, 1.36]
					rs6675281 Phe + rs821616 Cys-carriers ↑	
				Temporal CT	rs6675281 Leu/Leu ↓ rs6675281 Phe-carriers ↑	0.96 [0.38, 1.54]
				Parietal CT	n.s.	
				Occipital CT	rs6675281 Phe + rs821616 Cys-carriers ↑	
Suárez-Pinilla 2015^37^ PAFIP	CNR1 (a) rs1049353 (b) rs1535255 (c) rs2023239	Non-affective FEP *N* = 65 (rs1049353: 36 G/G; 29 A-carriers rs2023239: 49 T/T; 16 T/C) HC *N* = 12 (genetic analysis only)	2 scans (3 years)	Total GM volume Total WM volume	n.s.n.s.	
				CSF volume	n.s.	
				LV volume	n.s.	
				Thalamus volume	rs2023239: T/C patients ↓	0.59 [0.02, 1.16]
					T/T patients ↓	
					T/C patients ↓ >T/T patients	
				Caudate nucleus volume	rs1049353: Both A-carrier and G/G reduction was N.S. A-carriers 3.27 times greater ↓ than G/G patients.	0.76 [0.26, 1.27]
				CT	n.s.	
				Surface area	n.s.	
Addington 2005^39^ NIMH	GAD1 (a) 14 SNPs (b) one 4-marker combination	COS *N* = 72 (*N *= 39 with ≥ 2 scans)	2 scans (between 2 and 8 years)	Total GM volume	rs2270335 and rs2241165 were most strongly associated with progressive ↓	
				Frontal GM volume	rs2270335 and rs2241165 were most strongly associated with progressive ↓	
Hartz 2010^43^ Iowa longitudinal study of recent onset psychoses	G72 4-marker combination: rs3916965, rs3916967, rs2391191, rs778294. Hap 1: AGAG Hap 2: GAGG Hap 3: GAGA	Recent-onset SSD *N* = 110 [(37/57/16) had (0/1/2) Hap 1 alleles; (60/41/9) had (0/1/2) Hap 3 alleles] HC *N* = 120 (genetic analysis only)	At least 2 scans (mean: 3.0 (1.6) years)	Total cerebral cortex volume	Hap 1 had a significant effect on volume change	
				Total GM volume	n.s.	
				Total WM volume	Increased in Hap 3 homozygotes compared to other groups	1.31 [0.61, 2.01]
				Total frontal lobe volume	Greater ↓ in Hap 1 homozygotes than other Hap 1 groups. N.S. changes in other Hap 1 groups.	0.84 [0.30, 1.39]
				Frontal GM volume	Greater ↓ in Hap 1 homozygotes than other Hap 1 groups.	0.59 [0.06, 1.13]
				Frontal WM volume	Greater ↓ in Hap 1 homozygotes than other Hap 1 groups.	0.54 [0.01, 1.08]
					Hap 3 had a significant effect on volume change.	
				Total parietal, temporal, occipital volume	n.s.	
				Parietal, temporal, and occipital WM volume	Hap 1 had a significant effect on occipital WM change. Hap 3 had an effect on change in parietal WM, temporal WM, and occipital WM. Significant ↑ in Hap 3 homozygotes compared to other Hap 3 groups in all three regions.	
				Parietal, temporal, and occipital GM volume	Hap 1 had an effect on parietal GM change.	
		COS (onset before age 13) *N* = 83 (*N* = 61 with ≥ 2 scans) (29 Val/Val, 42 Val/Met, 12 Met/Met)			*COS and SIB:* Increased Val dose associated with accelerated CT thinning, particularly in fronto-temporal regions. HC: Increased Val dose associated with attenuation of CT loss in similar areas: bilateral PFC, temporal and superior parietal regions.	
Raznahan 2011^41^NIMH	COMT Rs4680 (Val158Met)	SIB *N* = 62 (*N *= 33 with ≥ 2 scans) (19 Val/Val, 30 Val/Met, 13 Met/Met) HC *N* = 208 (*N* = 141 with ≥ 2 scans) (57 Val/Val, 91 Val/Met, 60 Met/Met)	Variable, (approximately 2 years)	Cortical thickness	*COS* vs. *HC:* Significant differences in the relationship between Val dose and CT changes (i.e., accelerated thinning in COS and attenuated thinning in HC) were most prominent in left dlPFC and bilateral cingulate. Val dose-related CT decreases in dlPFC in COS compared to HC continued into adulthood.	
					*SIB* vs. *HC:* Significant differences in the relationship between Val dose and CT changes (i.e., accelerated thinning in SIB and attenuated thinning in HC) were most prominent in left IPS, bilateral IFG, AC and lateral temporal regions. Val-dose related CT decreases in SIB compared to HC resolved by age 20.	

*AAO* age at onset; *AC* anterior cingulate; *COS* childhood-onset schizophrenia; *CSF* cerebrospinal fluid; *CT* cortical thickness; *dlPFC* dorsolateral prefrontal cortex; *EES* estimated effect size, Cohen’s *d* with 95% confidence intervals calculated for significant findings based on data provided by the papers; *FEP* first-episode psychosis; *GM* gray matter; *Hap* haplotype, *HC* healthy controls; *IFG* inferior frontal gyrus; *IPS* intraparietal sulcus; *LV* lateral ventricles; *NIMH* National Institute of Mental Health; *NR* not reported; *n.s.*not significant; *PAFIP* Prospective Longitudinal Study on First-Episode Psychosis; *PFC* prefrontal cortex; *SCZ* schizophrenia; *SIB* siblings; *SSD* schizophrenia spectrum disorders (schizophrenia, schizophreniform disorder); *TBV* total brain volume; *WM* white matter.

^a^ For Addington et al.^40^ sample size for longitudinal neuroimaging data was variable at each time point. EES were thus based on the numbers of patients in each genotype group with baseline neuroimaging data available

#### BDNF

Out of four longitudinal G-NI studies evaluating BDNF, only one reported a significant association between BDNF variants and progressive brain changes. In an adult FEP sample, Met-carriers demonstrated significant progressive loss in frontal GM volume and progressive increase in LV and CSF volume over the three-year follow-up, while Val homozygotes did not show any significant changes during this period.^42^ No significant associations were observed between BDNF genotype and hippocampal volume change in either patients or HC in a FEP and HC study^34^ and a SCZ and HC study,^44^ or between BDNF genotype and changes in total brain volume (TBV), GM, WM, CSF, LV, thalamus and caudate nucleus volume in a FEP and HC study.^35^

#### NRG1

In an adult FEP sample, patient C allele carriers for SNP8NRG6221533 showed a significant increase in LV volume over 3 years compared to patient T homozygotes.^36^ Patient C allele carriers also showed decreased total WM volume compared to T homozygote patients at the 3-year time point, but the time x genotype interaction was not significant.^36^

In a second NRG1 study, family-based transmission disequilibrium tests (TDT) showed that a microsatellite (420M9-1395) was strongly associated with COS.^40^ COS risk allele carriers experienced a steeper rate of total GM volume loss into adolescence compared to COS non-carriers, a pattern observed throughout the temporal, parietal and occipital lobes. In the HC group, 420M9-1395 risk allele status affected GM and WM volume change in the occipital lobe only.

#### DISC1

Of two DISC1 SNPs tested in a 3-year follow-up study of adult FEP patients, rs6675281 (Leu607Phe) was associated with longitudinal changes in total, frontal and temporal CT.^38^ While patients homozygous for the Leu allele showed a significant progressive CT decrease in temporal cortex, patient Phe allele carriers showed a progressive increase in total, frontal, and temporal CT. A significant association between rs6675281(Leu607Phe) and rs821616 (Ser704Cys) combined genotypes was also observed, with increases in total, frontal and occipital CT found only in Phe + Cys-carrier patients.^38^

#### CNR1

Two of three CNR1 SNPs tested in a study in a FEP sample were associated with greater longitudinal decrease in volumes of subcortical structures.^37^ Caudate nucleus volume reductions over time were non-significant, but patient rs1049353 A-carriers demonstrated a 3.27 times greater decrease in caudate nucleus volume than G/G patients. For rs2023239, T/C patients showed a significantly greater longitudinal decrease in thalamic volume than T/T patients.

#### GAD1

Family-based TDT in COS patients and their parents indicated a significant association between 3 GAD1 SNPs (rs3749034, rs2270335, rs2241165) and COS phenotype. Quantitative TDT (QTDT) showed that two of these SNPs (rs2270335, rs2241165) demonstrated the strongest associations with longitudinal total and frontal GM volume loss of the SNPs tested. For both SNPs, the common alleles over-represented in COS were also the ones associated with greater GM volume loss.^39^

#### G72

Of three four-marker haplotypes representing G72 SNPs rs3916965, rs3916967, rs2391191, and rs778294, homozygosity for haplotype 1 (AGAG) was associated with greater total frontal volume loss over 3 years when compared to other haplotype 1 groups in a sample of recent-onset SSD patients. Homozygosity for haplotype 3 (GAGA) was associated with progressive increases in subcortical white matter volume across all lobes compared to other haplotype 3 groups.^43^

#### COMT

The COMT rs4680 (Val158Met) Val allele was over-represented in COS patients, and was associated with different patterns of CT development among COS, sibling (SIB), and HC comparison groups.^41^ Increased Val dose (having 2 vs. 1 vs. 0 Val alleles) was associated with an acceleration of cortical thinning in the COS and SIB group and an attenuation of CT loss in the HC group, particularly in fronto-temporal cortical regions. Differences were observed in the timing and regions most affected by Val dose in the COS and SIB groups (see Table 2).

### Association of G-NI results with measures of clinical severity and cognitive functioning

Three studies found an association between genetic variants linked with progressive brain changes and clinical or cognitive measures.^37,40,43^ In an adult recent-onset SSD sample, a four-marker G72 haplotype associated with increased frontal volume loss was also associated with greater severity of psychotic symptoms at follow-up (measured by the Scale for the Assessment of Positive Symptoms (SAPS) hallucinations and delusions domains).^43^ In a CNR1 FEP sample, rs2023239 was associated both with the rate of thalamic volume reduction and with the amount of improvement observed in positive and negative symptomatology during follow-up.^37^ Specifically, rs2023239 T/C carriers showed greater progressive decreases in thalamic volume as well as less improvement in positive (SAPS) and negative (SANS) symptoms than their T/T counterparts. Finally, in a COS cohort, possession of an NRG1 microsatellite (420M9-1395) risk allele was associated with steeper rates of GM volume loss, and this same risk allele was also associated with poorer premorbid functioning (as measured by the Premorbid Adjustment Scale).^40^

## Discussion

This is, to our knowledge, the first systematic review to explore the association between genetic variants and longitudinal structural brain changes in psychosis. Most of the genes yielding significant associations were evaluated in only one study (G72, DISC1, CNR1, GAD1, and COMT). Genes examined in more than one study were NRG1 and BDNF, and results from those studies were difficult to compare due to different genetic variants being tested (NRG1 studies) or considerable differences in demographic, clinical, and methodological variables (BDNF studies). A significant association between longitudinal volume or CT changes and specific genetic variants was most consistently reported in the frontal lobe, with five studies, out of seven specifically assessing this region, detecting a significant association. Results were mixed for the temporal, parietal and occipital lobes. This is consistent with meta-analyses usually reporting frontal regions as those showing the greatest longitudinal changes in studies measuring progressive brain changes in psychosis.^5,6,9^ Thus, the observation that genetic variants were primarily associated with changes in frontal regions may be linked with the increased vulnerability of this area to disease-associated longitudinal changes, particularly during the early phases of the illness and sensitive periods of neurodevelopment. Except for one study finding a significant association between polymorphisms in CNR1 and longitudinal changes in the caudate nucleus and thalamus, most of the studies assessing subcortical structures did not detect significant associations between genetic variants and changes in these regions. This is also consistent with meta-analyses of longitudinal brain changes in psychosis, which generally have not reported significant changes in subcortical structures over time,^45,46^ although one meta-analysis did report greater progressive decline in left caudate nucleus volume in SCZ patients compared to controls.^5^ In the studies yielding significant associations between genetic variants and longitudinal changes, effect sizes were usually of small or intermediate magnitude,^36–38,40,42,43^ with large effects (Cohen’s *d* ≥ 0.8) only found for change in total WM and frontal lobe volume in the study assessing G72^43^ and for change in total and temporal CT for the rs6675281 SNP in the study assessing DISC1.^38^ Since many genetic and environmental variables might impact deviant structural changes related to psychosis, it is to be expected that specific candidate genes may only account for very small amounts of variance in longitudinal brain changes seen in psychosis.

The most frequently studied gene among studies identified by our search criteria was BDNF. Structural alterations associated with BDNF Val66Met genotype were frontal lobe GM, LV and sulcal CSF volume in one recent-onset SSD study,^42^ but a separate study in non-affective FEP reported no interactions with these same regions despite similar cohort characteristics, length of follow-up, and image processing techniques.^35^ A possible explanation for the differing results may be the younger age at onset and shorter duration of illness in the recent-onset SSD study. Another explanation could be potential differences in AP treatment status at baseline and during follow-up between the samples, as antipsychotics may have an effect on brain changes and different genetic backgrounds may interact with that effect.^42^

The other gene examined in multiple longitudinal G-NI studies was NRG1, with results suggesting an effect for a 5’ SNP on LV changes in FEP patients and a 5’ microsatellite on the trajectory of brain changes in COS patients and controls.^36,40^ These two NRG1 studies and a COMT study were the only studies in this review to report that certain genetic variants were associated with greater progressive brain changes in patients but not in controls.^36,40,41^ These results suggest that certain genetic variants may have specific effects on progressive brain changes in psychosis. Another possibility is that certain variants have more marked effects on patients with psychosis, which make it easier to detect changes in these groups.

In the COMT study, findings in COS and SIB groups were similar during adolescence, but by early adulthood Val dose-related CT differences between SIB and HC groups had disappeared while Val dose-related CT differences between COS and HC groups remained. This suggests that CT alterations associated with COMT genotype may exist on a continuum between psychotic patients, their siblings, and healthy controls, with brain changes at certain developmental stages only detectable in the psychotic sample. These results underscore the importance of including comparison groups in G-NI studies, as effects of genetic variants on structural patterns may differ between patients, relatives, and controls. Longitudinal comparison of these groups could help identify at-risk phenotypes in addition to characterizing actual disease progression.

Along the lines of the COMT study, results from several other studies in this review suggest that the effect of certain genetic variants on brain changes might vary according to developmental stage or illness progression. NRG1 and DISC1 are highly relevant genes for neurodevelopmental and brain maturation processes. In the three studies examining these genes, cross-sectional results were either non-significant at some or all time points (as in the NRG1 FEP and DISC1 FEP studies),^36,38^ or only represented part of the developmental trajectory of volume changes (as in the NRG1 COS study).^40^ The effect of a CNR1 SNP on caudate nucleus reduction rate was also undetectable at cross-sectional analysis. These findings suggest that certain genes may affect the progression or trajectory of structural brain changes during the early years of illness and support the use of a longitudinal design to appropriately assess their association.

Three studies assessing genes involved in neurotransmission (GAD1, COMT, and G72) reported that genetic variants associated with progressive brain changes were also over-transmitted to patients or associated with the disease phenotype.^39,41,43^ Two 5’ GAD1 SNPs were associated with both COS and exaggerated cortical frontal GM loss.^39^ The Val allele at COMT Val158Met was overrepresented in COS patients (but not in their siblings) compared to controls, and was associated with accelerated CT thinning in patients. A G72 haplotype associated with increased frontal volume loss in a recent- onset SSD cohort ^43^ was consistent with alleles reported to be over-represented in SCZ cohorts in previous G72 genetic studies and meta-analyses.^47–49^ This G72 haplotype was also associated with more severe psychotic symptoms at follow-up.^43^ Aside from G72, genetic variants in two other genes (NRG1 and CNR1) were also associated with both progressive brain changes and clinical measures. The association of the same genetic variants with progressive brain changes, increased disease risk, and/or clinical variables point to their potential role in the pathophysiology of schizophrenia and other psychoses, and to alterations in brain structure as a core component of the illness. Future studies should further explore the links between genetic variants, brain changes, and clinical progression. A better understanding of these relationships may eventually contribute to increased accuracy in predicting disease course and greater individualization of clinical care in patients with psychosis.

Several limitations must be considered in interpreting the results of this review. First, the majority of the results have not been replicated, and since few studies evaluated a common genetic variant it was not possible to perform a quantitative synthesis and meta-analysis. Second, the overall number of studies included was low, all studies had relatively small sample sizes, and several of them were conducted by the same research groups. This means certain samples overlapped significantly and were comprised of participants from similar backgrounds that may not be representative of other populations. Considering studies carried out by different teams, several important factors such as cohort type, specific diagnosis, length of follow-up, ethnic composition, and genetic variant and NI measurement evaluated complicated direct comparison of results. Furthermore, besides those examining BDNF, almost every study reported a significant association between a genetic variant and progressive brain changes. This raises the question of whether associations with other brain regions may have been tested as well, but only positive associations were reported. Regarding the significance of findings, some of the studies did not provide sufficient information to calculate effect sizes and thereby quantify changes. Several of the studies did not include a healthy comparison group for NI changes. Another limitation was that cortical regions were assessed more often than subcortical regions during NI analysis, raising the possibility of region-reporting bias. Finally, most of the studies did not report on laterality when describing brain changes, making it difficult to determine whether changes were bilateral or whether any hemispheric differences were present.

In the studies that yielded significant results, potential confounding factors must be considered. AP treatment may have an effect on certain brain volumes,^19^ and these effects may differ depending on type (i.e., typical vs. atypical) of AP received.^50^ Research has suggested AP treatment may not only influence brain structure independently but also interact with genes evaluated in these studies, with animal studies showing different effects for atypical and typical APs on BDNF levels and cell proliferation in the hippocampus.^51,52^ Participants received different AP medications in several studies included in this review, but not all controlled for AP treatment in statistical analysis. Several other environmental factors may have affected results through interactions with genes, brain structure, or both, such as childhood trauma, stress, and cannabis use.^53,54^ For example, studies in cohorts of patients with psychosis have indicated interactions between childhood trauma, BDNF expression or genotype, and hippocampal volumes.^55–57^ The CNR1 study in this review reported interactions between CNR1 SNPs, and WM and LV volume changes in cannabis non-consumers, but not in cannabis consumers.^37^ Finally, given the limited variety of genes studied thus far, the lack of cohesive results may be partially due to the possibility that certain genetic variants relevant to progressive brain changes have not been studied yet. For example, genetic variants in genes related to inflammation and oxidative stress processes (such as genes involved in the synthesis and metabolism of glutathione, manganese superoxide dismutase, interleukins, major histocompatibility complex [MHC], or complement components) have increasingly been implicated in the pathophysiology of schizophrenia and other psychotic disorders,^58,59^ but are yet to be explored in a longitudinal G-NI study. Copy number variation (CNV) is another area that is yet to be extensively researched in relation to psychotic disorders. Certain copy number variants (such as 22q11.2 deletion, 16p11.2 duplication, and 3q29 deletion) have been implicated in the genetic architecture of psychotic disorders ^60–62^ and may be associated with structural brain differences in high-risk populations or patients with SCZ,^63,64^ suggesting these variants may be promising avenues of investigation in longitudinal G-NI research.

Candidate gene association studies in general are limited when used to address complex disorders and their associated outcomes, as significant associations are common but often fail to be replicated.^65^ While the candidate gene approach has had some successes in identifying genetic variants associated with complex brain diseases—one example being the association between *APOE* variation and Alzheimer’s disease risk and brain structure^66–68^—similar results have not been achieved in schizophrenia, and recent research using GWAS and meta-analysis suggests that many historical candidate genes for schizophrenia (including BDNF, COMT, DISC1, and NRG1) fail to reach genome-wide significance.^16,66^ One promising approach to address the issue of low reproducibility of candidate gene association studies is conducting large scale meta-analysis via consortia such as ENIGMA, which will likely play an increasing role in the identification and replication of genetic variants relevant to brain changes and psychiatric illnesses.^19,69^ Thus far, large-scale genetic research in schizophrenia has pointed to the extended MHC and several genes involved in glutamatergic synaptic function and calcium signaling are important loci for future research, and has also highlighted the potential value of polygenic risk score analysis.^16,17,66^ Even with these advances, individual studies remain important since large-scale analysis is impossible without the contribution of individual datasets, and certain hypotheses and causal questions may be better addressed, at least initially, in smaller studies.^69^

Despite the mentioned limitations, this paper represents the first systematic review and qualitative analysis of studies of genetic variants and progressive structural brain changes in psychotic cohorts. Results from this review suggest that several genes related to neurodevelopment, brain maturation processes, neurotransmission and plasticity might impact progressive structural alterations in psychosis. However, sparse studies and discrepancies in cohort characteristics, genes and specific variants examined and study methodology make forming substantial conclusions difficult. In future studies, research groups should attempt to reproduce results in larger samples and different populations so that replicability is established. Researchers should pay particular attention to consistency of design, genes evaluated, and imaging parameters used, so that a more cohesive literature base addressing the role of these factors in psychosis can be developed. Furthermore, an effort should be made to include longitudinal clinical and cognitive measures in order to increase the applicability of genetic neuroimaging findings to other facets of psychotic illness.

## Methods

### Search strategy

A systematic two-step literature search was performed according to the guidelines described in the Preferred Reporting Items for Systematic Reviews and Meta-analyses (PRISMA) statement.^70^ First, a PubMed database search from inception until November 2016 was performed with the following search terms: (gene OR SNP OR nucleotide OR polymorphism) AND (MRI OR “magnetic resonance imaging” OR neuroimaging OR cortical OR imaging OR DTI OR volume) AND (psychosis OR schizophrenia OR psychotic) AND (Humans[Mesh] AND English[lang]). Second, the reference lists of selected studies were manually reviewed to identify relevant studies missed by the initial computerized search. The original search yielded 1960 results, and five additional studies were identified during manual review.

### Selection criteria

1965 abstracts were assessed and considered for review if the study described met the following hierarchical eligibility criteria:

1. The study was published as an original peer-reviewed article written in English.
2. The study was performed in humans.
3. The study included a patient group with a diagnosis of schizophrenia or other psychotic disorders according to the Diagnostic and Statistical Manual of Mental Disorders (DSM) criteria (DSM-III, DSM-III-R, DSM-IV, DSM-IV-TR, DSM-5).
4. The study assessed both a genetic variant (SNP, microsatellite, or haplotype) and longitudinal changes in at least one structural and/or DTI-based MRI measure, including volume (total or regional cortical or subcortical brain volume, total or regional GM or WM volume, LV or cerebrospinal fluid [CSF] volume), cortical thickness (CT), cortical surface area (CSA), fractional anisotropy (FA), mean diffusivity (MD), radial diffusivity (RD), and axial diffusivity (AD).
5. The study had a longitudinal design, with participants undergoing at least two structural MRI assessments over a period of time.

**Fig. 1:**
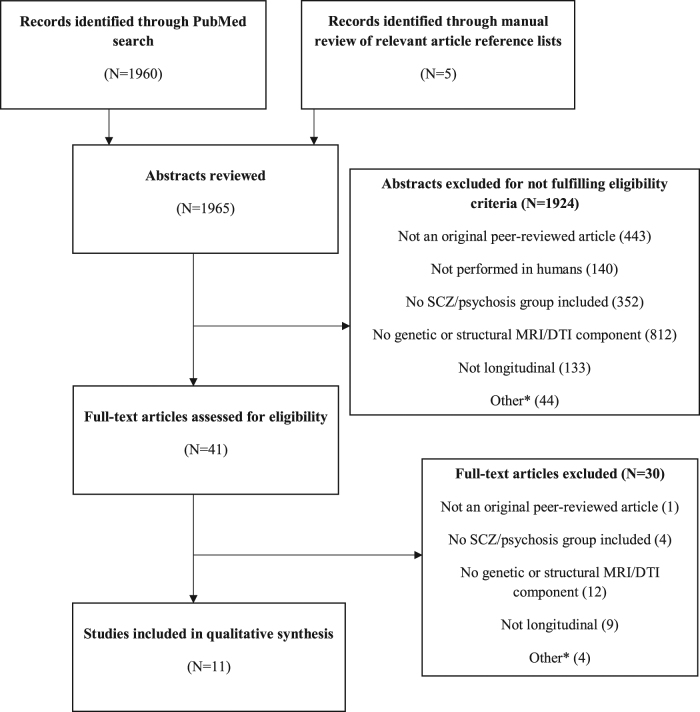
Flowchart of study selection. *Articles excluded for reasons not encompassed by other categories (*N *< 5; results had not yet been reported; psychosis was induced by medication or another biological syndrome; the topic of the article was not relevant*. SCZ s*chizophrenia;* MRI* magnetic resonance imaging; *DTI* diffusion tensor imaging

The full text was consulted in cases where information provided by the abstract was not sufficient to evaluate eligibility or where no abstract was available. After full text review, a final group of eleven studies using T1 weighted images were included in the review. Figure 1 provides a flowchart summarizing the selection process.

### Data extraction, synthesis, and analysis

Data for the following variables were retrieved for each study (see Supplementary Table 1): author names, year of publication, number of participants, diagnosis and diagnostic criteria, demographic variables (age at onset [AAO], age at baseline, duration of untreated illness [DUI], proportion of male subjects, medication, genetic data (gene and location; SNP, haplotype or microsatellite and location), structural neuroimaging data (number of scans, between-scan interval, structural brain measurements, software, scanner strength, type of T1-weighted sequence, slice thickness), genetic x neuroimaging outcome, and additional clinical or cognitive outcomes. Further information regarding genes and SNPs examined (i.e., function of gene, functional consequence of specific SNP) was collected from additional sources.^71–84^ For the purpose of synthesis and analysis, studies were grouped by gene examined and thereafter by year of publication. For individual studies providing sample size and relevant statistics for significant genetic x neuroimaging findings, estimated effect sizes (Cohen’s *d*) with 95% confidence intervals were calculated using Comprehensive Meta-Analysis Software version 3 (Biostat, Inc., Englewood, NJ).

### Quality assessment

The quality of the studies was assessed using an item-checklist designed specifically for this review based on a previously published quality assessment of longitudinal neuroimaging studies in psychosis.^45^ For each item, quality was assessed with a range of 0 (minimum) to 2 (maximum) points. The eleven studies were rated according to the sum of the points for each item, and then categorized as high (>80% of the maximum possible points), moderate-high (60–79%), moderate (40–59%), moderate-low (20–39%), or low (<19%) quality (see Supplementary Tables 2 and 3 for further details).

### Data availability

The authors declare that all data supporting the findings of this review are available within the paper and supplementary files.

## Electronic supplementary material

Supplementary Table 1

Supplementary Table 2

Supplementary Table 3
