# Supplementary material for: The association between gene variants and longitudinal structural brain changes in psychosis: a systematic review of longitudinal neuroimaging genetics studies
Source: NPJ Schizophr. 2017 Nov 1;3:40. doi: 10.1038/s41537-017-0036-2 (PMC5665946; doi:10.1038/s41537-017-0036-2)
Supplement: Supplementary file 1 — Supplementary Table 1 [file 41537_2017_36_MOESM1_ESM.pdf]

Supplementary Table 1: Additional characteristics and findings from the studies included in the review

Author, year, cohort, country (gene examined)	1) Duration of untreated illness (DUI)<br>2) Age at onset (AAO)	Age at baseline, sex	Diagnosis (diagnostic criteria)	Medication<br>1) % taking AP (FGA, SGA)<br>2) Exposure at baseline (mean ± SD) days<br>3) Dose during follow up (mean ± SD) mg	1) Type of image (type of sequence, slice thickness)<br>2) Voxel size<br>3) NI software	Additional NI results<br>1) Longitudinal NI results (patients)<br>2) Longitudinal NI results (patients vs. controls)
**Ho, 2007**<br>Iowa Longitudinal Study of Recent Onset<br>USA<br>(BDNF)	1) DUI: <5 years<br><br>2) AAO: Val/Val 24.3 (6.1) yrs, Met-carriers 24.8 (7.6) yrs	26.4 (6.8) yrs<br>83 (69.7%) male	Recent-onset SSD (DSM, CASH, entering within 5 years of onset)	1) Patients took AP for 84.9% of the follow up interval (mainly SGA)<br><br>2) 42% of patients AP-naïve at baseline<br><br>3) CPZ/day: (519.8 ± 472.6) mg	1) T1 (spoiled GRASS, 1.5mm), T2 and PD (3.0 or 4.0mm)<br><br>2) 1mm^3<br><br>3) BRAINS	1) NR<br><br>2) NA
**Koolschijn, 2010**<br>The Netherlands<br>(BDNF)	1) DUI: 12.7 (10.1) yrs<br><br>2) NR	SSD: 34.5 (11.4) yrs, 54 (79.4%) male<br><br>HC: 37.9 (13.5) yrs<br>56 (66.7%) male	SSD (DSM-IV, CASH)	1) Patients received FGA and SGA (most frequently SGA) AP prior to and during scan-interval<br><br>2) NR<br><br>3) NR	1) T1 (fast field echo, 1.2mm), T2 (dual echo turbo spin echo, 1.6mm)<br><br>2) 1 mm^3<br><br>3) Display (Brain Imaging Centre)	1) Hippocampal volume loss over time<br>2) Patients had smaller total, left, and right hippocampal volumes at baseline (F=5.102, p=0.025; F=4.031, p=0.046; F=5.171, p=0.024); HC group had greater total, left, and right hippocampal volume loss (ml change per year) than patients (F=11.091, p<0.001; F=9.939, p=0.002; F=9.035, p=0.003).
**Smith, 2012**<br>Canada<br>(BDNF)	1) DUI: Val/Val 13.0 months, Met-carriers 9.7 months<br><br>2) AAO: Val/Val 18.8 (6) yrs, Met-carriers 18.6 (5.8) yrs	FEP: Val/Val 20.6 (4.9) yrs, Met-carriers 20.6 (4.5) yrs<br>38 (65.5%) male<br><br>HC: Val/Val 22.4 (5.6) yrs, Met-carriers 19.9 (3.5) years<br>20 (51.3%) male	FEP (including affective psychosis) (DSM-IV)	1) 38% taking AP (100% SGA) at baseline; NR for follow up<br><br>2) 15 days (for 38% of cohort). 62% were AP naïve, 7% taking a mood stabilizer, 10% taking an antidepressant<br><br>3) NR	1) T1 (FSPGR IR prepped, 1.5mm)<br><br>2) 1.01mm^3<br><br>3) FSL (whole brain volume), FreeSurfer (hippocampus)	1) NS<br><br>2) NS
**Suárez-Pinilla, 2013**<br>PAFIP<br>Spain<br>(BDNF)	1) DUI: 28.3 (30.2) months<br><br>2) NR	FEP: 52 (65%) male<br><br>HC: 30 (55.5%) male	FEP (non-affective) (DSM-IV)	1) 100% (19% FGA, 81% SGA)<br><br>2) Baseline: (30.1 ± 25.1) days<br><br>3) NR	1) T1 (spoiled GRASS, 1.5mm), T2 and PD (3.0mm)<br><br>2) 1mm^3<br><br>3) BRAINS2	1) NR<br><br>2) NS differences

Author, year, cohort (gene examined)	1) Duration of untreated illness (DUI)<br>2) Age at onset (AAO)	Age at baseline, sex	Diagnosis (diagnostic criteria)	Medication<br>1) % taking AP (FGA, SGA)<br>2) Exposure at baseline (mean ± SD) days<br>3) Dose during follow up (mean ± SD) mg	1) Type of image (type of sequence, slice thickness<br>2) Voxel size<br>3) NI software	Additional NI results<br>1). Longitudinal NI results (patients)<br>2). Longitudinal NI results (patients vs. controls)
**Addington, 2007**<br>NIMH<br>USA<br>(NRG1)	1) NR<br><br>2) NR	COS risk allele carriers: 13.9 (2.7) yrs; COS non-carriers: 15.1 (2.4) yrs<br>46 (59.0%) male	COS (DSM-III-R or IV, onset of psychosis before age 12)	NR	1) T1 (spoiled gradient recalled echo, 1.5mm axial and 2.0mm coronal)<br><br>2) NR<br><br>3) NR	1) NR<br><br>2) NR<br><br>*Direct comparison between diagnostic groups was not possible due to the use of different statistical models for each group.
**Suárez-Pinilla, 2015**<br>PAFIP<br>Spain<br>(NRG1)	1) DUI: 30.5 (26.2) months (SNP8NRG221533 C-carriers), 31.1 (26.8) months (SNP8NRG221533 T/T)<br><br>2) AAO: 30.1 (8.5) years	FEP: 41 (69.5%) male<br><br>HC: 9 (64.3%) male	FEP (non-affective) (DSM-IV)	1) 100% (22% FGA, 78% SGA)<br><br>2) Baseline: (31.6 ± 27.6) days<br><br>3) Cumulative CPZ eq: (244,174.8 ± 159,729.7) mg	1) T1 (spoiled GRASS, 1.5mm), T2 and PD (3.0mm)<br><br>2) $1mm^3$<br><br>3) BRAINS2	1) LV volume changes (not further specified)<br><br>2) NA (no time or genotype effects in HC group; no genotype-by-time-by-case [patients vs HC] interactions observed)
**Vázquez-Bourgon, 2015**<br>PAFIP<br>Spain<br>(DISC1)	1) DUI: 26.2 (28.2) months<br><br>2) AAO: 29.5 (8.1) yrs)	49 (62%) male	FEP (non-affective) (DSM-IV)	1) NR<br><br>2) NR; no prior AP treatment or total lifetime treatment <6 weeks required<br><br>3) Cumulative CPZ eq: (220,741.2 ± 147,570.7) mg	1) T1 (spoiled GRASS, 1.5mm), T2 and PD (3.0mm)<br><br>2) $1mm^3$<br><br>3) BRAINS2	1) NR<br><br>2) NA

Author, year, cohort (gene examined)	1) Duration of untreated illness (DUI)<br>2) Age at onset (AAO)	Age at baseline, sex	Diagnosis (diagnostic criteria)	Medication<br>1) % taking AP (FGA, SGA)<br>2) Exposure at baseline (mean ± SD) days<br>3) Dose during follow up (mean ± SD) mg	1) Type of image (type of sequence, slice thickness<br>2) Voxel size<br>3) NI software	Additional NI results<br>1). Longitudinal NI results (patients)<br>2). Longitudinal NI results (patients vs. controls)
**Suárez-Pinilla, 2015**<br>PAFIP<br>Spain<br>(CNR1)	1) DUP: 13.8 (20.7) months<br><br>2) AAO: 29.9 (8.3) yrs	FEP: 44 (67.7%) male	FEP (non-affective) (DSM-IV)	1) 100% (22% FGA, 78% SGA)<br><br>2) Baseline: (31.9 ± 26.9) days<br><br>3) Cumulative CPZ eq: (234,641.5 ± 715,7361.6) mg	1) T1 (spoiled GRASS, 1.5mm), T2 and PD (3.0mm)<br><br>2) 1mm^3<br><br>3) BRAINS2	1) NR<br><br>2) NA<br><br>*Cannabis use by genotype interaction seen in non-consumer patients for rs1535255 and rs2023239.<br>Rs1535255: T-carrier non-consumer patients had a smaller total WM volume and a greater LV volume at baseline (WM: F=5.34, p=0.028; LV: F=5.00, p=0.033) and after 3 years (WM: F=12.85, p=0.001; LV: F=7.79, p=0.009) compared to C/C non-consumer patients.<br>Rs2023239: T/C non-consumer patients showed significant LV enlargement compared to T/T non-consumer patients after three years of follow-up (F=5.20, p=0.030).<br>NS differences between genotype subgroups in consumer patients.
**Addington, 2005**<br>NIMH<br>USA<br>(GAD1)	1) NR<br><br>2) AAO: 10 yrs	14.4 yrs<br>42 (58%) male	COS (DSM-III-R or IV, onset before age 12)	NR	1) T1 (Spoiled gradient recalled echo, 1.5mm axial)<br><br>2) NR<br><br>3) NR	1) NR<br><br>2) NA
**Hartz, 2010**<br>Iowa Longitudinal Study of Recent Onset Psychoses<br>USA<br>(G72)	1) DUI: <5 yrs<br><br>2) AAO: 22 (5.5) yrs	Recent-onset SSD: 26.7 (7.1) yrs<br>78 (71%) male	Recent-onset SSD (DSM-IV, CASH; entering within 5 years of onset)	1) 94%<br><br>2) 30% neuroleptic-naïve, 35% had received a low dose over the course of illness<br><br>3) CPZ daily eq (percentage of sample): 1-400mg (17%), 401-800mg 24%), >800mg (53%).	1) T1 (spoiled GRASS, 1.5mm). T2 and PD (3.0 or 4.0mm)<br><br>2) 1mm^3<br><br>3) BRAINS	1) NR<br><br>2) NA

Author, year, cohort (gene examined)	1) Duration of untreated illness (DUI)<br>2) Age at onset (AAO)	Age at baseline, sex	Diagnosis (diagnostic criteria)	Medication<br>1) % taking AP (FGA, SGA)<br>2) Exposure at baseline (mean ± SD) days<br>3) Dose during follow up (mean ± SD) mg	1) Type of image (type of sequence, slice thickness<br>2) Voxel size<br>3) NI software	Additional NI results<br>1). Longitudinal NI results (patients)<br>2). Longitudinal NI results (patients vs. controls)
**Raznahan, 2011**<br>NIMH<br>USA<br>(COMT)	1) NR<br><br>2) NR	COS: 14.6 (2.3) yrs, 48 (68%) male<br><br>SIB: 14.3 (3.9) yrs, 32 (52%) male<br><br>HC: 12.6 (2.9) yrs, 118 (57%) male	COS (DSM-III-R or IV, onset of psychosis before age 13)	NR	1) T1 (Spoiled gradient recalled echo sequence, 1.5 mm axial, 2.0mm coronal)<br><br>2) NR<br><br>3) CIVET	1) NR<br><br>2) NA

Abbreviations: AAO, age at onset; COS, childhood-onset schizophrenia; CPZ, chlorpromazine; eq, equivalents; DUI, duration of untreated illness; DUP, duration of untreated psychosis; FEP, first-episode psychosis; FGA, first generation antipsychotic; FSPGR, fast spoiled gradient echo; hap, haplotype; HC, healthy controls; LV, lateral ventricles; NA, not applicable; NIMH, National Institute of Mental Health; NR, not reported; NS, not significant; PAFIP, Prospective Longitudinal Study on First-Episode Psychosis; SCZ, Schizophrenia; SGA, second generation antipsychotic; SIB, siblings; SSD, schizophrenia spectrum disorders (schizophrenia, schizophreniform disorder).
