# Supplementary material for: The association between gene variants and longitudinal structural brain changes in psychosis: a systematic review of longitudinal neuroimaging genetics studies
Source: NPJ Schizophr. 2017 Nov 1;3:40. doi: 10.1038/s41537-017-0036-2 (PMC5665946; doi:10.1038/s41537-017-0036-2)
Supplement: Supplementary file 2 — Supplementary Table 2 [file 41537_2017_36_MOESM2_ESM.pdf]

Supplementary Table 2: Categories used for the quality assessment of the studies included in the review

Category / range	0	1	2
Funding sources	Not reported	-	Reported
Sample size[a]	<12	12-20	>20
Inclusion criteria	Not reported	Partly reported	Reported
Exclusion criteria	Not reported	Partly reported	Reported
Control group	Not included	Included for genetic analysis only	Included
Gender	Not reported	-	Reported
Race /ethnic origin	Not reported		Reported
IQ/ educational level	Not reported	Parental education reported	Reported
Duration of illness and/or age of onset	Not reported	-	Reported
Treatment with antipsychotic medication	Not reported	-	Reported
Drop-out rate	Not reported	-	Reported
Statistical values reported	Not reported	Mostly reported	Reported
Neuroimaging parameters[b]	Not reported	Partly reported	Reported
Genetic analysis protocol	Not reported	Partly reported	Reported

Max 28. ***High*** *(80-100%) >22,* ***moderate-high*** *(60-79%): 16.5-22,* ***moderate*** *(40-59%): 11-16.5,* ***moderate-low*** *(20-39%): 5.5-11,* ***low*** *(0-19%) <5.5*

[a] *Baseline sample size of patient genotype subgroups with both neuroimaging and genetic data available. One genotype group <12 + one genotype group >12 (0.5), one genotype group >20 + one genotype group 12-20 (1.5).*

[b] *Type of image, type of sequence, slice thickness, voxel size, NI software.*
