# Supplementary material for: The association between gene variants and longitudinal structural brain changes in psychosis: a systematic review of longitudinal neuroimaging genetics studies
Source: NPJ Schizophr. 2017 Nov 1;3:40. doi: 10.1038/s41537-017-0036-2 (PMC5665946; doi:10.1038/s41537-017-0036-2)
Supplement: Supplementary file 3 — Supplementary Table 3 [file 41537_2017_36_MOESM3_ESM.pdf]

Supplementary Table 3: Quality assessment and rating of the studies included in the review

Author, Year	Role of funding	Sample size	Inclusion criteria	Exclusion criteria	Control group	Gender	Race /ethnic origin	IQ/ educational level	DUI/ AAO	AP treatment	Drop-out rate	Statistical values reported	Neuroimaging parameters	Genetic Analysis Protocol	Sum of scores, category
Ho, 2007	2	2	2	0	0	2	2	2	2	2	2	2	2	2	24, High
Koolschijn, 2010	0	2	2	1	2	2	2	1	2	2	2	2	2	2	24, High
Smith, 2012	2	2	2	2	2	2	2	2	2	2	2	2	2	2	28, High
Suárez-Pinilla, 2013	2	1.5	2	2	2	2	2	0	2	2	2	2	2	2	23.5, High
Addington, 2007	0	2	2	1	2	2	2	2	0	0	2	2	1	2	20, Moderate-high
Suárez-Pinilla, 2015 (NRG1)	2	2	2	2	2	2	2	0	2	2	2	2	2	2	26, High
Vázquez-Bourgon, 2015	2	1	2	2	0	2	2	0	2	2	2	1	2	2	22, Moderate-high
Suárez-Pinilla, 2015 (CNR1)	2	1.5	2	2	1	2	2	0	2	2	2	2	2	2	24.5, High
Addington, 2005	0	2	2	1	0	2	2	2	2	0	2	2	1	2	20, Moderate-high
Hartz, 2010	2	1.5	2	0	1	2	2	0	2	2	2	1	2	2	21.5, Moderate-high
Raznahan, 2011	2	1.5	2	2	2	2	2	2	0	0	2	2	1	2	22.5, High

Abbreviations: AAO, age at onset; AP, antipsychotic; DUI, duration of untreated illness; IQ, intelligence quotient.
